# Health and social care staff’s experiences working with adults with complex needs – a focus group study

**DOI:** 10.1186/s12913-025-12770-1

**Published:** 2025-04-23

**Authors:** Ulrika Harris, Anna Andersson, Veronica Plessen, Markus Hjelm

**Affiliations:** 1 Blekinge Centre of Competence, Region Blekinge, Karlskrona, Sweden; 2 Department of Clinical Sciences in Malmö, Lund University, Lund, Sweden

**Keywords:** Adults with complex needs, Collaboration, Experiences, Integrated care, Multimorbidity, Participation, Psychiatric co-morbidity, Qualitative research

## Abstract

**Background:**

Multimorbidity is increasing globally, affecting over one-third of the population. Adults with complex needs often experience physical, mental, and cognitive disorders, leading to increased healthcare utilization, reduced quality of life, and social challenges. The frequent co-occurrence of psychiatric conditions, substance abuse, addiction, and homelessness highlights the complexity of these needs. Collaboration between healthcare and social services is essential for delivering integrated care but is often hindered by legislative constraints and difficulties in coordinating care. Although integrated care has been shown to improve outcomes, persistent challenges affect staff in their daily work with adults with complex needs. Therefore, the aim was to describe health and social care staff’s experiences working with adults with complex needs, with a focus on what promotes or hinders collaboration and the individual’s participation.

**Methods:**

This study employed a descriptive qualitative design. Data were collected through four focus group interviews with 17 health and social care staff members and analyzed using qualitative content analysis.

**Results:**

Data analysis resulted in three generic categories: (i) collaboration between authorities is complex, (ii) challenges working according to the person’s needs, and (iii) participation under difficult conditions.

**Conclusions:**

This study highlights both challenges and facilitators in working with adults with complex needs. Collaboration was hindered by legislative, financial, and organizational disparities but facilitated by interprofessional forums and collaborative meetings. Establishing trusting relationships free from bureaucratic constraints was important for providing person-centered care. However, fostering meaningful participation remains challenging because of the individual’s limited capacity to engage and the complexities that staff face in balancing respect for autonomy with acting in the person’s best interest. Further research incorporating perspectives from adults with complex needs, their relatives, and management could enhance the understanding of how collaboration, participation, and organizational barriers impact the provision of integrated healthcare and social services.

**Supplementary Information:**

The online version contains supplementary material available at 10.1186/s12913-025-12770-1.

## Background

The prevalence of multimorbidity is increasing worldwide and is estimated to be 37.2% of the total population [1]. Multimorbidity is commonly defined as the coexistence of two or more chronic diseases [2], including a combination of health problems such as mental health problems, cardiovascular diseases, metabolic diseases, and musculoskeletal disorders [3]. Multimorbidity is associated with increased healthcare costs and utilization, hospitalization, increased emergency care visits [4], decreased quality of life [5], and polypharmacy [3]. Adults with long-term mental, physical, and cognitive disorders have been shown to have a high disease burden and complex health needs [6], and individuals with severe mental illness are more than twice as likely to have physical multimorbidity [7]. Psychiatric conditions, harmful use, and addiction frequently cooccur in this group, indicating the need for more comprehensive treatment interventions [8]. These adults are frequently associated with homelessness and violence, often exhibiting a lack of insight into their condition and reluctance to accept assistance [9]. This highlights the importance of fostering integrated physical and mental health care [10]. The National Institute for Health and Care Excellence (NICE) [11] has defined adults with complex needs, which is in line with this study´s sample definition, as people aged 18 years or over who need a high level of support with many aspects of their daily life, and relying on a range of health and social care services. This may be due to illness, disability, broader life circumstances, or a combination of these factors [11]. Hereafter, this group is referred to as adults with complex needs. Adults with complex needs can experience cognitive difficulties, and when the care and support system fails to accommodate their vulnerable circumstances, they risk losing the motivation to seek help for their addiction or mental health issues [9].

Health systems often focus on individual conditions rather than multiple conditions, resulting in fragmented care and an increased treatment burden for adults with complex needs. For this group, person-centered care should be provided, with a focus on individual needs and values [12]. Interprofessional collaboration is essential to meet these individual needs, and collaboration between healthcare and social service providers is influenced by the degree of complexity in coordination and an individual’s capacity to participate and cocreate [13]. Adults with complex needs have the right to make informed decisions regarding their care [11]. However, this group can have a limited ability to collaborate [13]. Furthermore, they also need interventions from different authorities governed by different legislation, which hinders the flexibility required to create long-term solutions. The health system designed to support these individuals is perceived as complex, making it difficult for both service providers and individuals to navigate effectively [13, 14], risk leading to unmet care and support needs. In a study comparing 17 different integrated healthcare programs in Europe, good collaboration, patient participation, a holistic approach, and continuity were highlighted as important factors for improving the integrated care process for adults with complex needs [15]. Ivbijaro et al. [16] conclude that there is increasing evidence that a collaborative approach to delivering clinical care leads to improved health outcomes for individuals and communities.

In Sweden, national initiatives are underway to improve healthcare and social services for individuals with comorbid disorders, from both substance use or addiction and psychiatric diagnosis. These disorders require coordinated interventions across healthcare, social services, and addiction treatment. However, effective collaboration between providers is often challenging [9]. Further research is needed to explore how staff experience their work practices regarding adults with complex needs, to identify areas of improvement. Therefore, the aim was to describe health and social care staff’s experiences working with adults with complex needs, with a focus on what promotes or hinders collaboration and the individual’s participation.

## Methods

### Aim

The aim was to describe health and social care staff’s experiences working with adults with complex needs, with a focus on what promotes or hinders collaboration and the individual’s participation.

### Design

This study employed a descriptive qualitative design [17] with focus group interviews [18], as it facilitates conversation and encouragement among participants, which helps to uncover experiences and themes. The interviews were analyzed using qualitative content analysis [19] to gain a deeper understanding of the participants’ experiences.

### Setting

This study was conducted in one region of southern Sweden and adjacent municipalities. Healthcare and social services for adults with complex needs are available, and their provider responsibility is divided between the regional and municipal authorities. According to the Social Services Act (2001:453), municipalities are responsible for substance abuse and addiction care. This means that municipalities must ensure that individuals with substance abuse receive the assistance and care they need. Social services are also responsible for social support initiatives such as housing, employment, financial assistance, and support for children, relatives, and significant others. Regional authorities are obligated to provide medical care to all inhabitants within the region, in accordance with the Health and Medical Services Act (2017:30). This includes treatment for withdrawal symptoms, medication-assisted treatment for alcohol and drug abuse, and other medical and psychiatric interventions.

The social welfare board is responsible for applying for involuntary care due to substance abuse, regulated by the Act (1988:870), Care of Abusers, LVM. Adults may also be treated without consent under the Act (1991:1128) on Compulsory Psychiatric Care Act, LPT. Municipalities hold the primary responsibility for supporting individuals with substance use disorders, whereas regional authorities are required to provide medical services, including care for those involuntarily committed to LVM. The responsibility for adults with cooccurring disorders, in which substance abuse coincides with other psychiatric diagnoses, is distributed among multiple authorities. Both regional and municipal authorities make important contributions to the delivery of care and support services.

### Participants and data collection

Participants were recruited through purposeful sampling [17], as interest was in participants who could provide rich narratives of the study’s aim. The inclusion criteria were as follows: (i) employed as health or social care staff, and (ii) experience working in healthcare or social services involving adults with complex needs. Furthermore, a variation in regional or municipal authority employment was warranted to promote a variation of experiences to capture different perspectives relevant to the study´s aim.

Operational managers sent an information letter to potential participants with an invitation to participate in this study. If they were interested, they were invited to contact the research team by phone. The participants then received verbal information about the study, emphasizing that participation was voluntary and that they could withdraw from the study at any time without providing any reason. Twenty participants contacted the research team and were initially recruited for focus group interviews. However, three participants declined to participate because of illness or heavy workload, resulting in a final sample of 17 participants. The participants were then divided into four focus groups, with each group consisting of three to five participants. The number of focus group interviews was considered reasonable to answer the aim of the study, as indicated by previous research [20], and the interview sessions were rich in content. Prior to the interviews, all the participants provided written informed consent.

The four interview sessions were conducted by three of the authors (UH, AA and VP), who assumed the roles of the moderator (UH) and assistant moderator (AA or VP) [21]. The moderator’s task was focused on asking the interview questions and follow-up questions to the group, whereas the assistant moderator was helpful in solving practical tasks such as the monitoring of equipment and other practical issues. During the interview sessions, the moderator and the assistant moderator collaborated in their roles. A written and fictional patient case (Appendix 1) was constructed on the basis of feedback from a selected sample of health and social care staff with extensive experience in collaboration with adults with complex needs. These staff members were not included as study participants. Based on their experiences, a written case (Appendix 1) was constructed and used as the point of departure for the interviews. Before starting the focus group interviews, each participant was given time to read the patient case. A semistructured interview guide (see Appendix 2) was used to guide the interviews.

The interviews lasted between 101 and 122 min and were transcribed verbatim. The participants, comprising of women (*n* = 13) and men (*n* = 4), were between 31 and 63 years old and had 3–42 years of work experience.

### Data analysis

Transcribed interview materials from the four focus group interviews were analyzed using Elo and Kyngäs’s [22] inductive qualitative content analysis. Qualitative content analysis is a method that aims to obtain a condensed and broad description of the phenomenon. An inductive approach was selected because of the limited existing knowledge regarding the phenomena under investigation. The first step involved several thorough readings of the interview texts to become immersed in the data and to gain an overall understanding of its content. In the second step, the interview texts were coded by annotating and creating headings in the margins of the interview transcripts, guided by the aim of the study. In the third step, the codes were collated into a coding sheet. In the fourth step, codes were compared repeatedly to identify patterns of similarities and differences, leading to further abstraction and the development of subcategories and then generic categories. Finally, three generic categories and nine subcategories were identified. All the authors contributed to the data analysis in all the steps, with the author UH assuming primary responsibility for coordinating the analytical process. The authors worked collaboratively to ensure that no critical information was overlooked, and all the authors actively engaged in discussions regarding the findings until a consensus was reached.

## Results

In the analysis, health and social care staff’s experiences working with adults with complex needs were concluded in the three generic categories: (i) collaboration between authorities is complex, (ii) challenges working according to the person’s needs, and (iii) participation under difficult conditions, with nine included subcategories (see Table 1).

**Table 1:** Overview of generic categories and subcategories

Generic categories	Subcategories
Collaboration between authorities is complex	Knowledge of each other’s work responsibilities facilitates collaboration
	Challenges in collaboration between specialist and municipal care
	Tools for collaboration
Challenges working according to the person’s needs	Adapting communication based on the individual
	Strengthening motivation requires perseverance
	Coordinating to meet complex needs
	Unclear decision-making mandates
Participation under difficult conditions	Reduced ability for participation
	Acting in the best interest of the person

### Collaboration between authorities is complex

#### Knowledge of each other’s work responsibilities facilitates collaboration

The staff described having adequate knowledge of each other’s work responsibilities, utilizing each other’s competencies, showing respect, and maintaining close cooperation were all important aspects that facilitated collaboration between professionals. The staff experienced that when they got to know each other and gained more knowledge about the different organizations’ tasks and current legislation, it became easier to establish contact and collaborate on various interventions.

“You learn what you can do, where you have your own task… you get a greater understanding of each other’s professions. Because you always think, why do they not do more, and why is it so?” [Participant 2, group 2]

Continuity in collaboration was described as necessary to maintain and develop cooperation, which meant that structure, frameworks, and routines were needed; otherwise, collaboration would become person-dependent and unstructured. The staff described that collaboration forums, such as county-wide conferences, were important settings where different professionals could get to know each other and discuss topics from the staff’s perspective rather than focus on individual cases.

#### Challenges in collaboration between specialist and municipal care

The staff experienced various challenges in the collaboration between specialist and municipal care. Complicated referral procedures and slow processes negatively influenced their ability to provide tailored interventions for adults with complex needs. Another difficulty described was coordinating interventions and offering them at the right time, as different routines within each organization made this process time-consuming and difficult to address issues promptly.

The staff described that specialist care had specific tasks and that its responsibility for care ended once a patient completed the treatment. The responsibility was then transferred to the municipal authorities. However, these handovers were perceived as inadequate because of a shortage of staff and inpatient beds, which led to the early discharge of patients. Municipal care staff highlighted their own lack of psychiatric expertise and also described that psychiatric assessments were often missing for patients discharged from specialist care facilities. This situation led to individuals’ needs not being met, making it difficult to move forward with interventions. For example, placement in a residential treatment facility was perceived as an appropriate intervention provided that the person’s needs were updated and assessed in collaboration before, during, and after placement. However, a lack of collaboration, such as insufficient preparation, a lack of monitoring, and the absence of a structured care plan, negatively impacts the individual.

“Because they fall in between all the time. We see that we cannot do anything about the psychiatric issues because they take drugs or drink, and the social services cannot start treatment for the addiction because they feel mentally ill. And nobody…, and so we stand there and do nothing.” [Participant 2, group 1]

#### Tools for collaboration

The staff reported that they used tools for collaboration in their daily work, for example, to coordinate various interventions or follow care pathways. A central collaboration tool was the Coordinated Individual Plan (CIP), which was used to coordinate and plan efforts between different care providers and strengthen the individuals’ participation in their own care. CIP was described as useful for identifying a person’s needs, initiating or following up on interventions, and clarifying the roles and responsibilities of the professionals involved. In some cases, CIP was seen as demanding due to its rigid meeting structure, and staff members sometimes felt isolated in representing their professional roles during these meetings. It was perceived as effective when individuals felt safe, had sufficient time for reflection, and were motivated to participate in collaboration, but was considered more challenging when individuals struggled to express their own needs or when collaboration was lacking between professionals.

“Sometimes you have been in a really good CIP meeting, everyone walks away knowing what to do, you take that responsibility, we take this one, and you handle that, and this is how we will do it. And sometimes, you walk away like a question mark, feeling that this was just pointless.” [Participant 2, group 3]

Informal collaboration meetings were also described as useful for collaboration, in which the individual was not involved but provided consent for the meeting. These meetings allowed the staff to discuss a person’s needs and interventions. The staff expressed a need for such meetings, as they provided an opportunity to share thoughts and concerns without the individual being present. These meetings were considered important for building consensus and clarifying the responsibilities of the professionals. Without such preparatory meetings, CIP risked turning into a setting in which professionals blamed each other or created unrealistic demands. Therefore, the staff emphasized the importance of interprofessional collaboration before the CIP meeting to ensure a more constructive discussion. Another tool highlighted was the consultation teams between the region and municipality, where the staff had the opportunity to discuss de-identified cases, find solutions, and learn from each other.

### Challenges in working according to the person’s needs

#### Adapting communication based on the individual

The staff described the need to adapt their communication based on the individual’s circumstances, which required flexibility and trust. Being flexible in communication meant adjusting meeting times, locations, and methods according to the individual’s needs. A trusting relationship was considered essential, as individuals needed to feel safe enough to share their wishes and needs with the staff. The staff emphasized the importance of meeting places that promoted a sense of security, noting that an office environment was not always the most suitable setting. Suggested alternatives included an individual’s home or other locations perceived as neutral and relaxed, and it was considered important to allow individuals to choose their meeting place themselves. Some municipalities offered meeting points as a low-threshold intervention, where even individuals under the influence of substances were welcomed. This neutral environment facilitated communication and engagement.

The staff expressed that the persons often did not want to communicate via phone calls, but instead preferred SMS, email, or home visits. Some persons did not have a telephone or fixed address, which meant that home visits had to be conducted. It was important to tailor both the method and the level of information provided to ensure that it was perceived as relevant and accessible to the individual.

“Then it is important to determine whether the person can cope with listening to everything, or whether you should book another meeting. I… work differently for different individuals.” [Participant 2, group 2]

Staff emphasized that adapting communication required trust and highlighted that they needed to ensure that individuals felt safe expressing their wishes. Trust was also important for identifying needs and adapting communication in a reliable way. It was considered important that they discussed their perceived needs together, with the staff acting as a sounding board to provide explanations. This approach fostered an understanding of why the different interventions were provided.

#### Strengthening motivation requires perseverance

The staff experienced that strengthening a person’s motivation was a central part of their work and a long-term process that required perseverance. The ability to act quickly was necessary when a person showed motivation to change, as this was described as working within a narrow time window. To strengthen motivation, the work needed to be based on what the person wanted, which might start with small steps, such as agreeing to collect their medication once a week. The staff described that it was not possible to force someone but only to offer suggestions. To do this effectively, both the staff and the person needed to regularly acknowledge even small improvements to sustain motivation.

“Because then you will see this window … it does not have to be once a year, it can be several times, it is always short-lived, but if you meet the person more often, it is of course more likely that you will encounter this window more often.” [Participant 1, group 1]

Motivational work was often perceived as demanding, involving many repetitive processes that took time and required perseverance. It was important not to lose commitment when other interventions were initiated or when a person’s condition deteriorated. It was also important to seize the temporary window of opportunity when a person’s motivation was high and to act quickly to facilitate change before the opportunity passed. However, the staff described this as challenging because they were dependent on the actions of others, and these efforts did not always happen as quickly as needed.

#### Coordinating to meet complex needs

In their work, the staff found it essential to coordinate interventions between multiple actors over longer periods of time to be able to meet the persons’ complex needs, but this collaboration was often lacking. The staff expressed the need for a coordination function to support them in their work. For example, seeking and maintaining housing for people with substance abuse is an intensive and lengthy process that requires coordination to support the housing process. There were examples of coordinating functions such as permanent care contact, but this required an already established connection with psychiatry. Other functions, such as personal representatives, guardians, and trustees, could also coordinate efforts, but there were differences in how the scope of these tasks was interpreted and implemented in practice.

The staff expressed a great need for a case manager function that could assess the whole picture and help guide adults with complex needs through various healthcare and social service contacts. A case manager’s function was described as requiring a comprehensive understanding of the unique situation of adults with complex needs, as well as knowledge of relevant legislation at both the regional and municipal levels, to guide individuals effectively to appropriate measures.

“If this person needs supported housing, their own housing, or special housing… just that someone guides them there, to apply for the right intervention, that can be a great help because we have several people who do not take that step or do not know what is available or do not know what they want.” [Participant 1, group 4]

#### Unclear decision-making mandates

Staff experienced that their ability to work effectively was hampered by aspects such as long decision-making paths, unclear decision-making mandates, and complicated processes. This led to decisions taking a long time, as many cases needed to be investigated and examined, which became a problem since the persons often needed interventions immediately. The staff expressed that the decision-making process meant that many managers needed to be involved, which made the process more complex.

Staff described that previous organizational changes had resulted in decisions and mandates being transferred to senior managers rather than remaining with those working closest to the individuals. High staff turnover further complicated the decision-making process, and the staff expressed that they were losing momentum and constantly had to start over. They expressed a desire to place responsibility on one authority rather than on several authorities, as was the case at the time. Such organizational change would have prevented individuals from being repeatedly transferred between regional and municipal services.

“As cost-saving measures have been implemented, mandates have moved upward. So, things that I could decide to myself 20 years ago I cannot do today, because it has to… not even my boss can, but her boss has to decide. And in 99.9% of cases, it ends with the same decision I would have made from the start.” [Participant 2, group 1]

### Participation under difficult conditions

#### Reduced ability for participation

The staff described that they met adults with complex needs who had a reduced ability to function in everyday life, manage a home, manage finances, communicate adequately, and live up to society’s requirements. These individuals were often perceived as lacking sufficient self-awareness of their problems, which meant that they did not recognize the same problems identified by the staff. For example, limited self-awareness could make it more difficult for a person to participate and affect their receptiveness to various support measures. This created frustration among the staff when they rejected the proposed support measures and did not want to be involved, which in turn made it more difficult to achieve change. This situation caused staff to lose the motivation to continue working on the cases, as they began questioning the effectiveness of the interventions when the same individuals repeatedly returned for support.

“Many have no insight themselves, and they are not really… it becomes difficult to reach them. They either refuse or sometimes do not have the ability to accept what is offered.” [Participant 5, group 1]

#### Acting in the best interest of the person

Staff experienced that the persons they met lacked trust in the authorities and had experienced repeated failures, making their hope for change fragile. The staff expressed that all interventions were voluntary and that the persons must not be forced, regardless of whether a high need for intervention had been identified. Even if an individual did not want to participate, it was important to maintain contact and offer support. The staff described that their work was about striking a balance between demanding that the person took responsibility and being aware of the person’s inability to fully assume this responsibility. They emphasized the importance of being honest with the person about what was required of them in certain support interventions while simultaneously assessing the person’s ability to succeed in making a change.

“Of course, you want the participation to be there and for the person to want that. Is it okay that I contact the psychiatry? So, you want a yes there… To collaborate and go forward, so it’s not just about going over someone’s head, like, you said no, but I am doing this anyway.” [Participant 1, group 4]

Sometimes, relatives acted in the person’s interest without the individual’s participation. The staff described that, in such work situations, they walked a tightrope between respecting the person’s own will and simultaneously having to act against the person’s will, with the motive that they had to act in the person’s best interest and move forward in the decision-making process.

## Discussion

The main results of this study are presented in the following three generic categories: (i) collaboration between authorities is complex, (ii) challenges in working according to the person’s needs, and (iii) participation under difficult conditions. The findings highlight the importance of recognizing and planning for challenges that arise in collaboration between regional and municipal authorities, in providing healthcare and social services, and in supporting adults with complex needs to participate in their care.

The results illustrate that collaboration between professionals within the involved authorities is challenging and complex, leading to slow and unclear processes that could negatively impact the care of adults with complex health needs. This is further challenged by the authorities’ diverse legislation, financial systems, care structures, and working methods. Leijten et al. [23] developed the SELFIE framework, which can be used to describe, develop, implement, and evaluate integrated care programs for multimorbidity. This framework emphasizes that, while organizational and structural integration can enhance integrated care, policies ensuring service availability and access are also essential. The findings from our study indicate that staff members experienced difficulties working within a fragmented health system, which was perceived as an obstacle to providing sustainable, person-centered solutions for adults with complex needs. This aligns with Almqvist and Lassinantti [24], who found that professionals aim to promote empowerment and build strong relationships with clients, but are constrained by an inflexible health system. They concluded that the complexity lies not with the clients but with the health system they must navigate. Hence, our study highlights the need for continuity and sustainable structures to support collaboration between the involved authorities, a challenge also identified by D’Amour et al. [25]. Consequently, fostering strong professional relationships and building trust through ongoing formal and informal communication, such as regular information meetings and collaborative forums, may contribute to more sustainable collaboration [15, 25].

Our findings illustrate that promoting individual participation among adults with complex needs remains a challenge because of their vulnerable life situations and reduced ability to be involved in their care. The findings highlight that staff must balance a person’s needs with the needs they, as professionals, consider essential for a sustainable life situation. The SELFIE framework [23] emphasizes that managing multimorbidity requires prioritization, tailored care, and shared decision-making among providers, caregivers, and the individual. Knutsson and Schön [26] stressed the importance of recognizing clients as partners and promoting cultural and normative shifts at all levels of the health system to enhance collaboration around individual needs. Previous studies [26–28] from a Swedish context have illustrated staff ambivalence toward patient participation in care planning. While they strive to foster relationships and encourage participation, they also feel obligated to follow organizational interests and routines [26–28]. Consequently, participation has, to some extent, been taken over by staff who guide and steer individuals toward the right path in managing their needs.

Our findings highlight the need for improved coordination between healthcare and social services for adults with complex needs. The staff reported feeling constrained by the lack of a mandate to act, limiting their ability to address gaps in care. One proposed solution was the implementation of a case manager function, which could enhance the continuity of care and facilitate regular contact with individuals in need [15, 24]. Despite the Swedish National Board of Health and Welfare’s [29] recommendation to provide case management services for individuals with cooccurring disorders, most healthcare and social service organizations do not offer such support in Sweden. Furthermore, the findings also emphasize the importance of multidisciplinary and flexible teams, as collaboration remains challenging within a complex system shaped by both organizational structures and individual factors. According to D’Amour et al. [25], collaborative approaches often fail when key components, such as establishing shared goals and visions across all organizational levels, are missing. Furthermore, staff identified missing components for integrated care but lacked a mandate to address them, as many aspects fall under the management’s responsibility. To strengthen integrated care, Looman et al. [30] suggested that organizations must actively support professionals coordinating integrated care, such as care coordinators and case managers. Consequently, organizations, networks, and local governments should appoint alignment workers to bridge different levels of care, translate policies into organizational change, and influence the broader health system to accelerate integration. This highlights the need for structural reforms to empower staff, promote interdisciplinary collaboration, and establish clear governance frameworks to enhance the delivery of integrated care.

### Methodological considerations

This study followed the Consolidated Criteria for Reporting Qualitative Research (COREQ) guidelines to ensure methodological rigor [31]. This study discusses trustworthiness in accordance with Lincoln and Guba’s criteria, including credibility, confirmability, dependability, and transferability [32]. Purposeful sampling was used to enhance the credibility. This sampling technique involves identifying and selecting individuals or groups who have extensive knowledge and experience related to the phenomenon of interest [33], that is, the experience of working with adults with complex needs.

Data were collected through four focus-group interviews with 17 participants, which provided information-rich discussions and generated interview data suitable for data analysis. The data analysis process was rigorous and involved triangulation and active participation from all the researchers. Reflective and critical discussions were conducted by the research team throughout the data analysis process to strengthen confirmability and ensure that the interpretations were grounded in the data. Quotations from the participants were used to illustrate the findings and enhance transparency. Transferability was addressed by providing detailed descriptions of the research context, participant characteristics, and data collection procedures.

One limitation of using focus group interviews is the potential difficulty of capturing individual or opposing perspectives [18]. Although all participants worked with adults with complex needs, they were employed by different regional or municipal authorities, which may have influenced their willingness to discuss sensitive issues related to each other’s organizations. However, according to the interviewer and moderator, this did not seem to limit the discussion; rather, the participants engaged openly and addressed several challenges. Another limitation is the gender distribution of the participants as only four were male. Given that most staff members in the included sectors of healthcare and social services in Sweden are female, this reflects the general workforce composition. Nevertheless, the limited male representation may affect the transferability of the findings.

## Conclusions

Our findings highlight both challenges and facilitators in health and social care staff’s experiences working with adults with complex needs. Disparities in legislation, financial systems, care structures, and organizational cultures created barriers to collaboration, while interprofessional forums and coordinated individual plan meetings supported care coordination and multidisciplinary engagement. The staff emphasized the importance of fostering trust and relationships with the individual, that are, free from bureaucratic constraints, to provide person-centered care. However, enabling meaningful participation in decision-making remains challenging because of the individual’s limited capacity to engage and the complexities that staff face in balancing respect for autonomy with acting in the person’s best interest. Further research incorporating perspectives from adults with complex needs, their relatives, and management could enhance the understanding of how collaboration, participation, and organizational barriers impact healthcare and social services, and the provision of integrated care.

## Supplementary Information

Supplementary Material 1.

Supplementary Material 2.

## Data Availability

Data excerpts are provided in the manuscript. The qualitative data collected in this study are not publicly accessible, given the challenges in ensuring complete anonymity. The participants did not provide consent for public sharing of their data.
