# Supplementary material for: Health and social care staff’s experiences working with adults with complex needs – a focus group study
Source: BMC Health Serv Res. 2025 Apr 23;25:583. doi: 10.1186/s12913-025-12770-1 (PMC12016296; doi:10.1186/s12913-025-12770-1)
Supplement: Supplementary file 1 — Supplementary Material 1. [file 12913_2025_12770_MOESM1_ESM.docx]

**Appendix 1**

**Case**

Person 30 years old who has been to a treatment center for mixed addiction about 20 times and several times at the psychiatric intensive care unit and at the psychiatric emergency department. The person has received interventions sporadically from social services, mainly for addiction that has existed since adolescence. The person has a suspected psychotic disorder, but only want medical treatment for his ADHD and does not want any other contact with psychiatry.

The person's housing situation has been unstable and has involved moving back and forth between different municipalities and even outside the country. The person has been evicted and has been homeless for periods. The person has temporarily lived with his mother but is no longer welcomed there. Now, only want help to get a place to stay in another municipality. There is a threat to the person from criminal networks linked to debts. The person has been assigned a guardian for some time and the guardian is responsible only for finances.

The person has previously behaved violently towards other people in town and has also attacked staff at a ward in a treatment center and was at that time discharged. The last time the person sought care at the health center was due to poor mental health, and was referred to the emergency ward. The person was then admitted to the hospital in connection with the emergency visit, but declined and only wanted medication for ADHD. An assessment was made that the patient did not meet the criteria for LPT. A report of concern was reported to the municipality under Section 6 LVM because of ongoing substance abuse, but the person expressed no need for help. Later, social services contacted the person based on the notification received. Afterwards, the person applied for help with housing and support to get ADHD medication and agreed to a Coordinated Individual Plan (CIP).
