# Supplementary material for: Health and social care staff’s experiences working with adults with complex needs – a focus group study
Source: BMC Health Serv Res. 2025 Apr 23;25:583. doi: 10.1186/s12913-025-12770-1 (PMC12016296; doi:10.1186/s12913-025-12770-1)
Supplement: Supplementary file 2 — Supplementary Material 2. [file 12913_2025_12770_MOESM2_ESM.docx]

**Appendix 2**

**An interview guide**

The participants are provided with practical information on how the focus group interviews will proceed.

The group interview starts with the participants reading the fictious case and individually reflecting on it for a few minutes.

The interview starts with the open-ended question: Please tell me what are your thoughts on this case?

**Follow-up questions that can be used when needed**

Please tell me, how would you choose to handle this case?

Tell me, what are the chances of success, and what are the risks of acting in one way or another?

What challenges/opportunities do you identify in this case?

Please tell me, what does participation mean to you?

What can promote or impede the individual’s participation?

What conditions exist in the organization that can impact the individual's participation in the design of care and support?

Please tell me, in what ways do you collaborate internally/externally in your organization in the work with adults with complex needs?

Tell me, what promotes/impedes collaboration in the work with adults with complex needs?

Please tell me, is there a common understanding of ways of working with adults with complex needs?

Do you experience that you have sufficient knowledge of your collaboration partners in the work with adults with complex needs?

Do you experience that different organizations and parties' regulations affect the work with adults with complex needs?

Do you experience that you have the knowledge and support you need to work with adults with complex needs?

Good examples - when has it worked? Tell me, have you experienced any long-term, sustainable solutions?

Follow-up questions, such as "Could you give me an example?" and "Can you tell me more?", will be asked as needed throughout the interview to further explore and deepen the topics that emerge during the interview.
